# Community Perceptions of Executive Orders Impacting Lung Cancer Care, Research, and Mental Health

**DOI:** 10.3389/ijph.2025.1608714

**Published:** 2025-11-12

**Authors:** Shanada Monestime, Bradley Newton, Joelle Fathi, Danielle Hicks, Lori Millner, Laurie Ambrose, Elridge Proctor, Chaya Estrin-Lebovic, Courtney Granville

**Affiliations:** GO2 for Lung Cancer, Washington, DC, United States

**Keywords:** lung cancer, executive orders, community perceptions, mental health, cancer policy

## Abstract

**Objectives:**

In January 2025, federal executive orders introduced sweeping changes to healthcare, public health infrastructure, and research funding. These shifts raised concern within the lung cancer (LC) community, which depends on early detection, timely care, and continued innovation. This study examined community perceptions of these policy changes.

**Methods:**

We conducted a mixed-methods national survey (March 2025) guided by the Social Ecological Model. A 12-item online survey collected responses from individuals with personal or professional ties to LC. Descriptive statistics and inductive thematic analysis were performed.

**Results:**

Among 497 respondents, 239 met inclusion criteria. Most reported a personal (69.5%), professional (20.0%), or combined (10.5%) connection to LC. Over half cited emotional distress, disrupted care, and research-funding concerns. Thematic analysis of 81 responses revealed six themes: fear and uncertainty; reduced access; research loss; and mixed views, with some supporting or opposing the executive orders. Patients and caregivers most often reported fears regarding Medicaid cuts, delayed diagnostics, and stalled innovation.

**Conclusion:**

Findings highlight emotional, operational, and research-related disruptions perceived by LC communities and reinforce the urgency of centering affected voices in health-policy decisions.

## Introduction

Lung cancer remains the leading cause of cancer-related deaths in the United States, with underserved communities continuing to face persistent challenges related to delayed diagnoses, limited treatment options, and inequitable access to care [1–3]. Recent orders may further threaten access to quality care in underserved rural and urban areas by reducing telehealth services, imposing hiring freezes that exacerbate staffing shortages, disrupting the supply chain for essential equipment, and cutting budgets for community health programs. In recent years, meaningful progress has been made across the prevention and care continuum, including primary prevention efforts through smoking cessation and tobacco control, secondary prevention via early detection through low-dose CT screening, tertiary prevention through advancements in detection of residual disease and application of biomarker-driven therapies, and availability of clinical trials [4–8]. These advancements have collectively contributed to improvements in lung cancer survival.

Sustaining this momentum depends on stable public health infrastructure, robust research funding, and supportive health policy environments [9]. As seen during COVID-19, the lung cancer community, which includes patients, caregivers, clinicians, and researchers, remains especially vulnerable to systemic disruptions that may hinder access to life-saving innovations or continuity of care, particularly in settings such as the Veterans Health Administration and other federally supported health systems [10–13]. Building on those lessons, a new wave of federal actions introduced additional uncertainty when the Trump administration introduced a series of executive orders that led to immediate changes in U.S. healthcare and research policy [14, 15]. These actions included halting new grants and contracts, limiting the operational capacity of federal agencies, and withdrawing funding from collaborative health initiatives [14, 15]. Key institutions such as the National Institutes of Health and the Centers for Disease Control and Prevention faced new restrictions on research activities, staffing, and external engagement [16, 17]. The administration’s withdrawal from the World Health Organization (WHO) further compounded concerns regarding the future of scientific collaboration and global health coordination [14, 18].

Public health leaders, clinicians, researchers, and patients have expressed deep concern about the impact of these executive orders on both research continuity and access to quality care [19, 20]. Freezing billions in research investments and destabilizing agency operations risks delaying treatment breakthroughs, slowing clinical trial enrollment, and reducing access to guideline-directed care, including smoking cessation programs, telehealth services, and lung cancer screening support in already underserved communities [19, 21, 22]. For individuals affected by lung cancer, policy shifts of this magnitude may introduce new barriers to diagnosis, treatment, and participation in emerging research [14, 19].

Although these policy changes have received national and international attention, there remains limited empirical data on how they are both perceived and experienced, directly and indirectly, by those most impacted. In particular, the perspectives of those directly engaged in the lung cancer community, whether personally or professionally, are largely absent from current literature. To address this gap, GO2 for Lung Cancer (a national patient advocacy organization) conducted a community-based survey in March 2025. This manuscript presents an exploratory analysis of the policy-related experiences of individuals across the lung cancer care continuum, with the goal of informing future advocacy, policy response, and programmatic support.

## Methods

### Study Design, Sampling, and Survey Development

This study used a mixed-methods approach to explore how recent executive orders may be affecting individuals in the lung cancer community. A 12-item survey was developed to collect both quantitative and qualitative data and was administered through SurveyMonkey, a secure online platform, between 3 March and 8 March 2025. The survey was designed to take fewer than 10 minutes to complete and included both closed-ended questions (e.g., role, affiliation, familiarity with policies) and open-ended prompts to capture qualitative responses. The response options “Industry Partner” and “Other” were added to the affiliation question on 4 March 2025, after ∼180 responses had been submitted. This update was made in response to early participant comments and emails noting that industry-affiliated respondents did not have an appropriate category. Consequently, only later participants could select these options (Link to survey: https://go2.org/eo-survey/).

The study was designed to capture real-time community perceptions rather than achieve statistical inference. A convenience sampling approach was employed, leveraging GO2 for Lung Cancer’s national digital network to engage individuals across the lung cancer care continuum, including patients, caregivers, clinicians, researchers, and advocates. Due to the study’s exploratory mixed-methods design, which prioritized inclusivity and breadth of perspectives over probabilistic sampling, the sample size was determined by the total number of complete and valid responses received during the open survey window.

### Participant Recruitment and Eligibility

Participants were eligible if they self-identified as being personally impacted by lung cancer (such as patients, caregivers, or family members) or professionally engaged in the field (including clinicians, researchers, industry affiliates, advocates, or policy stakeholders). Participation was voluntary, and responses were anonymous unless participants opted to share contact information for follow-up. Individuals were recruited through GO2 for Lung Cancer’s national network using email lists, social media platforms, and professional contacts across the lung cancer field. This recruitment strategy engaged a broad, advocacy-connected segment of the lung cancer community, providing insights from those most attuned to ongoing policy and research developments. At the start of the survey, participants were asked a screening question: “Do you work in the field of lung cancer, or have you been personally impacted by lung cancer?” Individuals who selected “No” were automatically excluded from participation by the survey platform, serving as the study’s primary exclusion criterion. These respondents were included in the total number of survey starts but not in the analytic sample. Based on early participant feedback, the response options “Industry Partner” and “Other” were added on March 4, 2025, after approximately 180 responses had already been submitted, limiting how many participants could select these categories.

### Data Analysis

All survey data were reviewed and cleaned to ensure completeness and validity. Responses that did not meet the inclusion criteria or were duplicate entries identified by identical content and IP address were excluded from the analysis. The final analytic sample was organized into two categories: respondents with personal connections to lung cancer and those with professional roles in the field. Quantitative responses were analyzed descriptively to summarize respondent characteristics and perceptions of policy impact.

Open-ended responses were analyzed using inductive Thematic Analysis to identify and interpret patterns of meaning across participants’ narratives. This approach was selected for its flexibility and suitability for exploratory research aimed at understanding perceptions and experiences without imposing predefined categories. A line-by-line coding approach was used to identify emergent ideas, which were then organized into broader thematic categories aligned with the levels of the Social Ecological Model (SEM), a framework that examines how individual behaviors and outcomes are shaped by interactions across multiple levels of influence (e.g., individual, interpersonal, organizational, community, and policy) [23, 24]. Applying the SEM in this way allowed for a more integrated interpretation of how experiences at each level intersected to shape community perceptions of policy impact.

## Results

### Baseline Characteristics

A total of 497 individuals responded to at least one survey item. After applying exclusion criteria, 239 responses were retained for analysis. Of these, 166 (69.5%) reported a personal connection only to lung cancer (e.g., as patients, survivors, or caregivers), 48 (20%) reported a professional connection only (e.g., clinicians, researchers, or advocates), and 25 (10.5%) reported both personal and professional connections to lung cancer. Table 1 presents a detailed breakdown of participants’ roles and their relationship to lung cancer.

**TABLE 1:** Respondents’ relationship to lung cancer (United States, 2025).

Overall Relationship to Lung Cancer^a^ (N = 239)	n (%)
Personal connection	166 (69.5%)
Professional relationship	48 (20%)
Both personal and professional	25 (10.5%)

Individual Roles^a^	n (%)
Patients	149 (62.3%)
Caregivers	24 (10.0%)
Family member or friend impacted	42 (17.6%)
Healthcare provider	42 (17.6%)
Healthcare administrator or leader	12 (5.0%)
Industry representative	3 (1.3%)
Community leader or advocate	19 (7.9%)
Policymaker	1 (0.4%)
Researcher	10 (4.2%)

^a^ Each respondent was assigned to only one group—personal, professional, or both—based on their overall combination of roles. For example, a participant who identified as both a caregiver and healthcare provider was categorized as “both personal and professional.”

**FIGURE 1:**
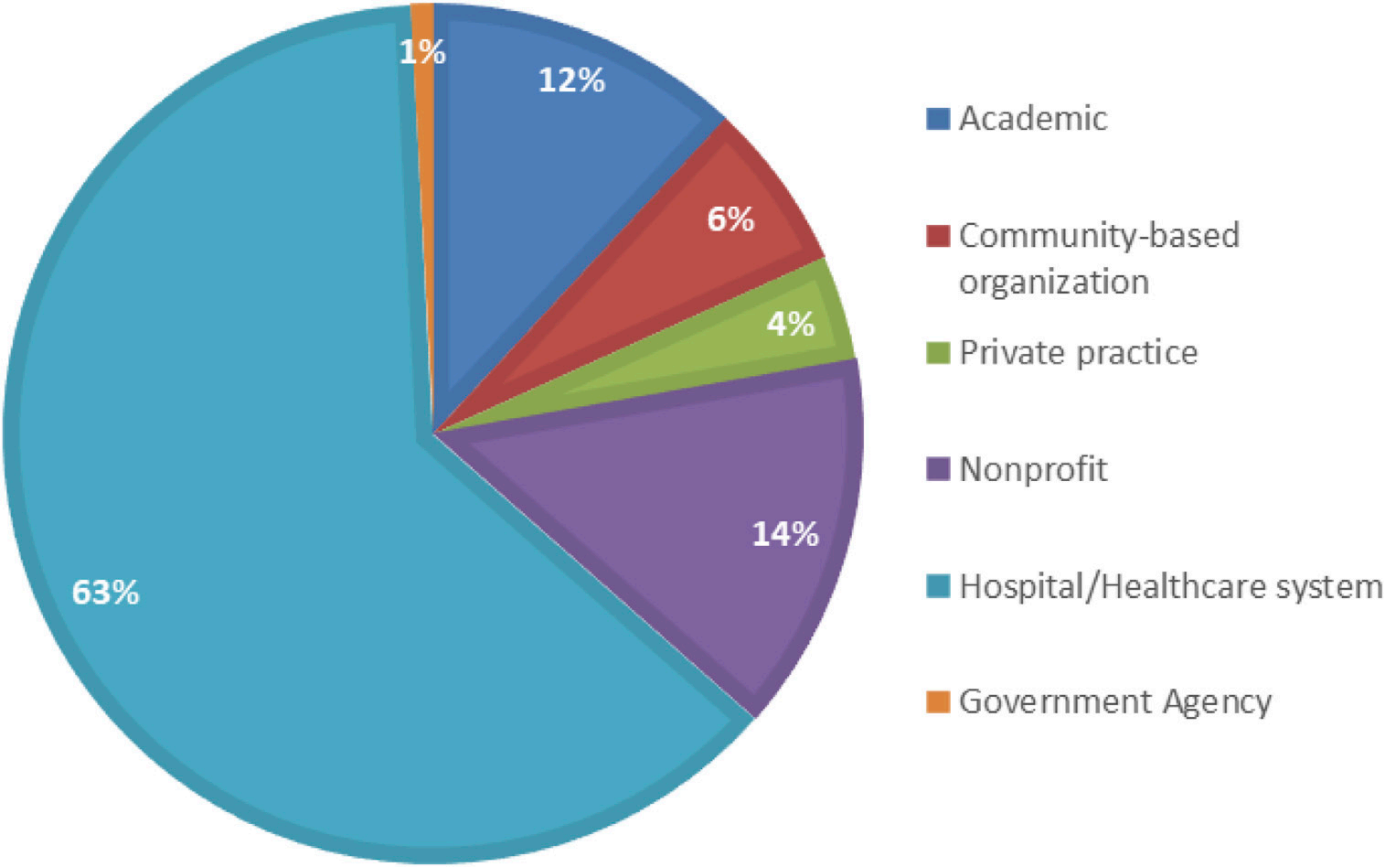
Primary lung cancer–related work setting or affiliation (United States, 2025).

Approximately half of the respondents reported no formal affiliation with a specific type of institution or organization. Among those who did report an affiliation, hospitals or healthcare systems were the most commonly cited (63%, n = 79), followed by nonprofit organizations (14%, n = 18) and academic institutions (12%, n = 15). Affiliations with community-based organizations, government agencies, and private practices were less frequently reported. The distribution of work settings and affiliations is presented in Figure 1.

### Awareness and Perceived Impact of Executive Orders

**FIGURE 2:**
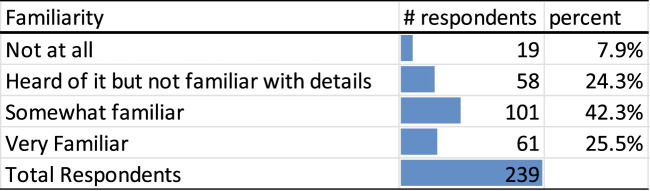
Familiarity with recent executive orders impacting healthcare and research (United States, 2025).

**FIGURE 3:**
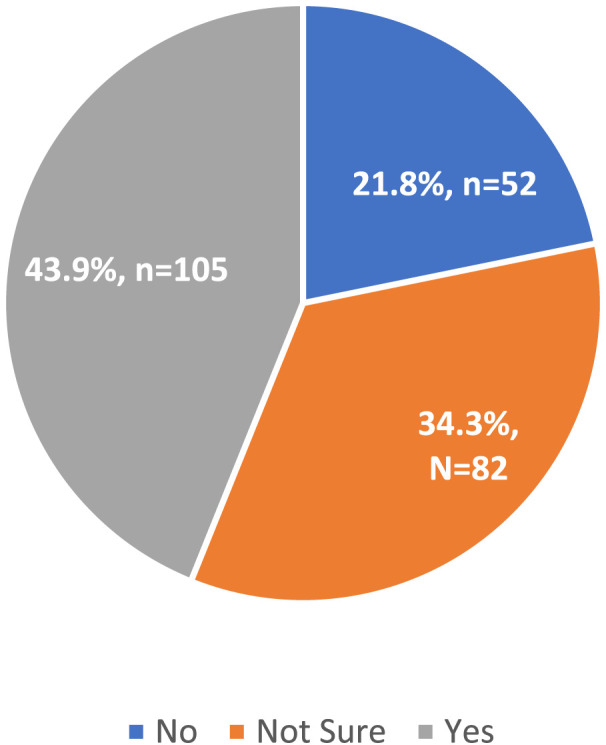
Belief that the executive orders have impacted respondents’ work or daily life (United States, 2025).

The vast majority of respondents (67.8%) indicated that they were at least somewhat familiar with the recent executive orders affecting healthcare and research (Figure 2). When asked whether the executive orders had impacted their personal or professional lives, the majority of respondents (43.9%) indicated they were experiencing an impact. However, 34.3% remained unsure about whether the policies would affect them, and 21.8% reported no perceived impact (Figure 3).

### Specific Areas of Impact

When asked to identify specific areas already affected by the executive orders, more than half of the respondents (55%) reported that their emotional or mental wellbeing had been negatively impacted. In addition to emotional strain, respondents reported experiencing disruptions to research funding, access to care, job security, and insurance coverage. Notably, 15% of respondents cited a negative impact on their ability to seek funding for new research and on the availability of community-based resources. Approximately 19% of respondents indicated that none of the listed areas had been impacted. A breakdown of response frequencies is provided in Table 2.

**TABLE 2:** Reported areas affected by recent executive orders on healthcare and research (United States, 2025).

Impacts	Number	Percent Overall
Ability to provide patient care	16	7%
Availability of medications or treatments	22	9%
Job security or funding for your work	25	10%
Healthcare coverage or insurance policies	29	12%
Access to healthcare services	32	13%
Interruption of current clinical trial or research	32	13%
Access to clinical trials or research opportunities	34	14%
Ability to seek funding for new research opportunities	36	15%
Community resources and support systems	36	15%
None of these	46	19%
Emotional or mental wellbeing	132	55%
Total Respondents	239	-

### Perceived Significance of Policy Impact

**FIGURE 4:**
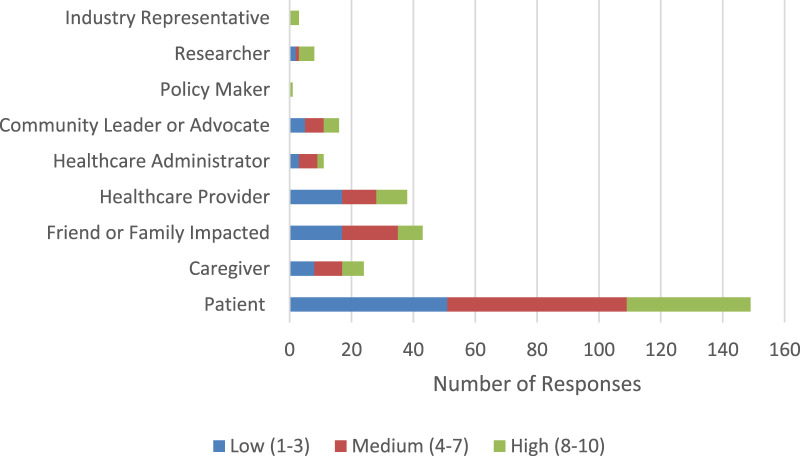
Perceived significance of impact among respondents by role (United States, 2025).

Patients represented the largest respondent group, with 34% rating the significance of the executive orders’ impact as low (1–3), 39% as medium (4–7), and 27% as high (8–10). Friends or family members impacted by lung cancer followed, with 37% reporting low impact, 39% medium, and 17% high. Healthcare providers reported 41% low, 27% medium, and 24% high. Among caregivers, 38% rated the impact as low, 43% as medium, and 19% as high. A more detailed breakdown of responses across additional roles can be found in Figure 4.

### Interest in Continued Engagement

A total of 106 (44%) respondents expressed interest in participating in a follow-up interview to further share their experiences. This high level of engagement indicates a strong willingness among members of the lung cancer community to monitor and continue discussing how federal policy changes are affecting their care, work, and overall wellbeing.

### Qualitative Results: Thematic Analysis

A total of 81 (33%) participants provided open-ended responses describing how recent executive orders may be influencing their personal lives, work, or communities. Of these, 59 (72.8%) had personal backgrounds (e.g., patients or caregivers), 11 (13.6%) had professional backgrounds (e.g., healthcare providers, researchers, or advocates), and 11 (13.6%) reported both personal and professional experience. Thematic analysis, guided by the SEM model, revealed six key themes reflecting experiences across multiple levels of influence, including individual factors such as fear, anxiety, and uncertainty; interpersonal and community factors such as challenges in healthcare access and disruptions to caregiving and support systems; and policy-level factors such as research funding loss, as well as varying levels of support for or opposition to the executive orders. These themes reflect diverse emotional responses, policy considerations, and political viewpoints, illustrating how individuals in the lung cancer community are interpreting and processing the potential implications of recent policy changes.

### Fear, Anxiety, and Uncertainty

The theme of *fear, anxiety, and uncertainty* was the most prevalent, reported by 23 (28.4%) respondents. Participants expressed worry about the future, fear about losing access to care, and emotional distress tied to the uncertainty surrounding federal policy changes. Many noted that the psychological toll was significant—especially for those already coping with a cancer diagnosis or caring for loved ones.

“The fear mongering has definitely made an impact. We are all afraid of what’s to come.”

“The emotional health and wellbeing of my spouse, friends, and relatives has been severely negatively impacted by these executive orders.”

### Healthcare Access

Sixteen (19.8%) open-ended responses focused on anticipated or experienced challenges accessing healthcare services. These included fears of losing Medicaid, delays in diagnostic services, inability to afford medications, and concern over reduced support at treatment centers. Participants who relied on safety-net programs or lived in rural communities were especially vocal in this category.

“I am nervous if my cancer is to return that I will not have the same access to care or specialists. I am also afraid of cuts to Medicaid that will affects scans, lab work, etc …”

“Beyond executive orders, diminishing Medicaid funding will severely negatively impact our ability to care for rural patients.”

“I am also treated at the VA. Because of both the cuts and the hiring freeze, simple procedures like dental visits for routine exams and cleanings have gone from a normal 6-month rotation to, now, an 8.5-month rotation. Every employee received the “fork in the road” memo, and those who took it are not being replaced. Probationary and temporary employees are gone, and the hole they leave behind is not being refilled. Equipment is not being recalibrated or repaired because many of the people that did that work are no longer around. So, services to veterans are getting delayed, rescheduled, or cancelled altogether.”

### Research Funding Loss

Fourteen responses (17.3%) raised alarms over reduced federal investment in lung cancer research. Participants pointed to cuts to NIH funding, canceled clinical trials, and slowed progress toward treatment development. For many, this theme represented not only a professional concern but also a personal fear tied to survival and innovation.

“Reducing NIH funding and preventing the best universities in America and scientists from doing research … will set back cancer care and innovation in devastating ways.”

### Stable or Unaffected Status

Eleven participants (13.6%) reported that the executive orders had not yet had a direct effect on them personally or professionally. However, their responses often reflected a tone of caution or anticipation, suggesting that many were bracing for future disruptions.

“Again no impact yet … but expect there to be some issues down the road.”

“Too soon to say the magnitude of changes but the future impact could be a 10.”

### Support for Executive Orders

Nine responses (11.1%) expressed approval of the executive orders. Supporters described the changes as necessary, overdue, or beneficial for bringing oversight and accountability to the healthcare system. These participants generally viewed the orders as a correction to systemic inefficiencies.

“I’m very glad to finally have an administration that is providing oversight and not just allowing every bad actor to thrive off cancer patients.”

“Positively. Fully support President Trump. These actions are long overdue”

### Opposition to Executive Orders

Eight responses (9.9%) reflected strong disapproval of the executive orders or the administration enacting them. Respondents voiced frustration with the direction of federal policy, concerns about marginalized populations being left behind, and fear that years of progress in lung cancer research and care were being undone.

“This administration is destroying years of research findings and treatment and should be held accountable …”

## Discussion

The ongoing implementation of executive orders affecting healthcare and scientific research has raised significant concern across multiple sectors [14, 20]. This study sought to understand how these policies are perceived and experienced by individuals in the lung cancer community, whether through lived experience or professional engagement. The survey aimed to fill a gap in the current understanding of how broad federal actions are being interpreted and experienced on the ground, especially within a community already facing disproportionate health burdens. While much of the national discourse around executive orders focuses on administrative and fiscal impacts, this study was needed to explore the personal and professional implications for those navigating cancer care and research on a daily basis.

One of the most novel findings of this study was the perception of halted or delayed clinical trials following the executive orders. Respondents, especially those in professional roles, described delays or cancellations of clinical trials, reduced grant availability, and broader instability in the cancer research ecosystem. These reports point to concrete fears that policy-related disruptions could waste ongoing research investments, interrupt patient enrollment, and stall scientific progress, consistent with broader reports on the consequences of restricting funding to institutions such as the National Institutes of Health, which supports the majority of cancer research in the United States [19]. Such disruptions not only threaten access to innovative treatments but also undermine trust in the scientific process and confidence in the stability of ongoing research. Even in the early months following the executive orders, respondents perceived a slowdown in research momentum, suggesting that policy instability alone can generate apprehension that affects participation in ongoing studies and the willingness of patients or institutions to engage in future trials. Some participants also reported disruptions to healthcare services, and community-based resources as a result of the executive orders. However, a subset of participants reported no current impact, either because they had not yet experienced any direct disruptions or because they were unsure how the policies would affect them. These varied responses point to differences in exposure, timing, and personal context.

Another central theme that emerged was the emotional and psychological impact of the executive orders. More than half of respondents, across various roles, reported that their emotional or mental wellbeing had already been affected. Patients and caregivers described heightened anxiety and fear about losing access to treatment, medications, or clinical trials, with some expressing concern that halted research could threaten their future care or survival. They believed that cuts to Medicaid or federally funded screening programs would delay diagnoses and worsen outcomes, while uncertainty about available support services and limited communication further heightened their frustration. Participants’ experiences build on existing research showing that individuals affected by lung cancer already face significant emotional strain and limited support networks [25]. The added uncertainty surrounding these policy changes may further heighten psychological stress, which is known to influence cancer outcomes by elevating stress hormones that promote inflammation, suppress immune response, and support tumor growth and progression [26]. However, this uncertainty not only impacts patients, but for professionals, this instability can create concern about job security and halt innovation. Although emotional impact is not always the primary focus of policy analysis, it is an important indicator of community-level distress and perceived vulnerability, particularly among those managing life-threatening illness [27]. These findings parallel observations in other public health contexts where federal policy shifts have created widespread uncertainty and behavioral change among affected populations. For example, qualitative research conducted following major immigration policy changes found that fear, reduced trust in institutions, and avoidance of healthcare settings were common reactions among communities facing sudden political and regulatory shifts [28]. Such parallels reinforce that policy instability can generate cascading psychosocial effects across diverse populations. Recognizing these cross-cutting dynamics highlights the need for proactive communication and stabilization efforts during periods of policy transition to mitigate unintended harm.

Support and opposition to the executive orders were nearly equal among respondents. Some viewed the actions as necessary to improve oversight and address inefficiencies in the healthcare system. Others strongly opposed the changes, expressing concern that they could reverse progress in lung cancer care and further disadvantage marginalized communities. These contrasting perspectives reflect the polarizing nature of recent federal actions and highlight the importance of including community voices when evaluating policy impact. While this survey did not stratify results by race, geography, or socioeconomic status, it is reasonable to expect that such disruptions—if sustained—could have a greater impact on populations already facing barriers to care. Differences in perception may also stem from underlying personal and contextual influences. Individual attitudes toward healthcare and policy are shaped by a complex interplay of intra- and interindividual factors, including emotional states, prior experiences with care, and sociocultural context. Recent conceptual work emphasizes that these variables may moderate or mediate how individuals perceive and respond to health-related changes, influencing trust, engagement, and perceived vulnerability within healthcare systems [29]. Recognizing this complexity provides a more nuanced understanding of our findings, as participants’ emotional and cognitive responses to policy shifts likely reflect broader personal, social, and informational environments.

This study has several limitations. As a brief online survey distributed through GO2 for Lung Cancer’s network, the sample may reflect higher engagement levels among individuals already involved in advocacy, support communities, or digital platforms. As a result, the perspectives of people with limited access to online tools or those outside of established lung cancer networks may not be fully represented. Additionally, demographic variables such as age and education were not collected, which limits the ability to assess how these characteristics may have influenced participants’ perceptions, emotional responses, or attitudes toward healthcare. Political affiliation was also not collected, which could represent a potential confounder given the policy context. However, responses reflected a nearly even balance of support and opposition to the executive orders, suggesting that perspectives were diverse and not dominated by one ideological stance. Additionally, there may be selection bias, as individuals who felt emotionally impacted by the executive orders may have been more motivated to participate. Finally, because this was a cross-sectional study, it captures perceptions at one point in time and may not reflect evolving views or downstream effects of policy changes.

Future research should explore the long-term impact of these executive orders, particularly how they affect research funding, clinical trial access, and patient outcomes. Our qualitative findings also highlight opportunities for deeper exploration through follow-up interviews with participants who expressed interest in continued engagement, which could provide richer insights into how perceived policy uncertainty translates into measurable effects on care delivery, research continuity, and emotional wellbeing within the lung cancer community. It may also be valuable to pair perception data with actual healthcare utilization and research productivity metrics, which would allow policymakers and advocates to better assess the real-world implications of federal policy shifts. Building on this, rapid survey like ours can serve as an early-signal mechanism to identify community concerns in real time, providing timely evidence to inform policy discussions and advocacy priorities. Such rapid feedback approaches are particularly valuable when policy changes evolve faster than traditional research cycles can capture their effects. Future iterations could also incorporate perspectives from policymakers and agency stakeholders to bridge community feedback with decision-making processes.

### Conclusions

This study offers early insight into how recent federal actions are perceived and experienced across the lung cancer community, including patients, caregivers, clinicians, and researchers. Findings revealed emotional distress, disrupted clinical research activities, funding instability, and concerns about reduced access to critical resources, highlighting the real-time effects of policy changes. While some participants supported certain aspects of these policies, the range of perspectives shared in this study highlights the importance of developing policies that reflect the real-world needs and experiences of those affected by lung cancer. Future directions should ensure that federal decisions support continued progress that guarantees access to high-quality cancer screening and care for everyone in need.
